# An intelligent humidity sensing system for human behavior recognition

**DOI:** 10.1038/s41378-024-00863-6

**Published:** 2025-01-22

**Authors:** Huabin Yang, Qiming Guo, Guidong Chen, Yuefang Zhao, Meng Shi, Na Zhou, Chengjun Huang, Haiyang Mao

**Affiliations:** 1 Institute of Microelectronics of the Chinese Academy of Sciences, Beijing, 100029 China; 2 University of Chinese Academy of Sciences, Beijing, 101408 China; 3 BYD Auto Industry Company Limited, Shenzhen, 518118 China

**Keywords:** Nanoscale materials, Sensors

## Abstract

An intelligent humidity sensing system has been developed for real-time monitoring of human behaviors through respiration detection. The key component of this system is a humidity sensor that integrates a thermistor and a micro-heater. This sensor employs porous nanoforests as its sensing material, achieving a sensitivity of 0.56 pF/%RH within a range of 60–90% RH, along with excellent long-term stability and superior gas selectivity. The micro-heater in the device provides a high operating temperature, enhancing sensitivity by 5.8 times. This significant improvement enables the capture of weak humidity variations in exhaled gases, while the thermistor continuously monitors the sensor’s temperature during use and provides crucial temperature information related to respiration. With the assistance of a machine learning algorithm, a behavior recognition system based on the humidity sensor has been constructed, enabling behavior states to be classified and identified with an accuracy of up to 96.2%. This simple yet intelligent method holds great potential for widespread applications in medical assistance analysis and daily health monitoring.

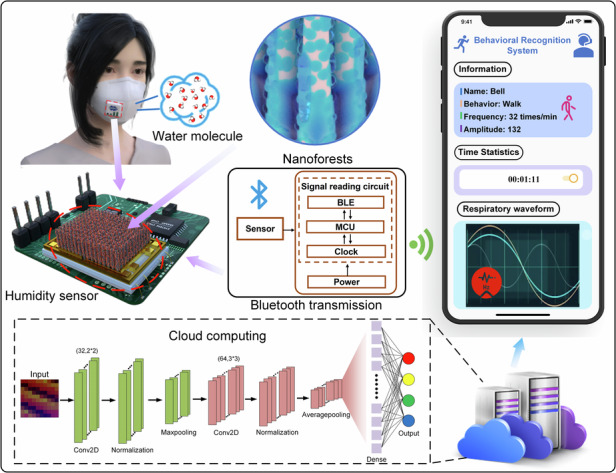

## Introduction

The development of intelligent sensing technology has enabled the recognition of human behaviors by distinguishing both physical and physiological information^1–5^. Behavior recognition has demonstrated great promise in various applications, such as smart homes and healthcare. In smart homes, behavior recognition can optimize residents’ comfort by adjusting appliances and monitoring the safety of children and the elderly based on their activities^6–8^. For patients with specific diseases requiring sufficient exercise or the avoidance of vigorous activity, behavior recognition can trigger alerts when their conditions do not meet these requirements^9–11^. This technology can also detect and analyze the daily emotions of specific groups, helping to identify negative emotions and prevent emotional depression^12–14^. Therefore, advancing human behavior recognition may yield significant benefits for intelligent living and the auxiliary medical industry, warranting further investigation.

Various approaches have been developed for human behavior recognition, with the most widely adopted relying on extracting physical information from video, analyzed using algorithms^15–20^. However, the efficiency of visible light imaging is limited by environmental factors, such as inadequate lighting, necessitating complex algorithms to enhance accuracy. Furthermore, fixed camera placements limit operability and raise privacy concerns. A wearable approach to behavior recognition often requires smart devices integrated with sensors that identify body activities based on heart rates (detected by pressure sensors) and the amplitude and frequency of arm movements (utilizing accelerometers, gyroscopes, and altimeters)^21–23^. The necessity of multiple sensors for comprehensive information collection increases the system’s size, cost, and data processing complexity.

Respiration is a vital physiological function facilitating gas exchange between the body and the surrounding environment. The frequency and intensity of breathing vary with different physical states, which allows for the inference of behavioral states from respiratory characteristics^24,25^. Consequently, there is a growing demand for sensors capable of detecting behavior based on respiration^26^. Notably, human respiration originates in the lungs, where the relative humidity (RH) of exhaled airflow can exceed 90%. This fact creates opportunities for developing humidity sensors for respiration detection. To date, there has been significant attention on humidity sensors designed to respond to the humid airflow present during respiration^27–30^. While exhalation produces highly humid airflow that increases the sensor’s output signals, inhalation results in external airflow that displaces water molecules from the device, thereby reducing RH and diminishing output signals. It’s important to recognize that daily life encompasses a range of behaviors, some of which occur rapidly with subtle respiratory differences. Thus, high sensitivity and rapid response are crucial for humidity sensors employed in this context. In particular, situations characterized by anxiety or fear may lead to weak breathing airflow, necessitating the development of extremely sensitive devices to detect such behaviors.

Current humidity sensors are constrained by the materials used in their construction, resulting in insufficient sensitivity. Moreover, many of these devices rely on differences in signal frequency to assess respiratory states, which limits their capacity to recognize a broader spectrum of behaviors^31–33^. These sensors typically consist solely of interdigital electrodes (IDEs) and sensing materials and fail to address temperature drift issues in signal acquisition, presenting a significant challenge for humidity sensors. Such issues can lead to deviations in respiratory waveforms across varying behaviors and potentially impact the accuracy of behavior recognition. Furthermore, most research on behavior recognition using humidity sensors primarily relies on simulated data, and the recognition results are not thoroughly reported^34,35^.

In this work, we propose a highly sensitive humidity sensor that utilizes nanoforests (NFs) as the sensing element. This development aims to create a high-accuracy system capable of intelligently recognizing human behaviors. In addition to employing NFs as the sensing material, the sensor incorporates a micro-heater to maintain high operating temperatures, further enhancing sensitivity. A thermistor with excellent linearity is also integrated into the sensor to accurately monitor the temperature of exhaled airflow, allowing for precise temperature compensation. Based on the superior performance of the humidity sensor, we have developed a data processing method to synthesize one-dimensional signals—namely humidity, temperature, and time—into three-dimensional maps. Consequently, our system achieved a remarkable accuracy rate of 96.2% for wireless real-time human behavior recognition, anticipated to find applications in smart home and health management domains.

## Materials and methods

### Materials

Photosensitive polyimide (PI, ZKPI-810) was purchased from POME Technology Co., Ltd., China. Carbon dioxide (CO_2_, 10,000 ppm) and nitrogen (N_2_, 99.999%) were obtained from North Special Gases Co., Ltd., China. Oxygen (O_2_, ≥99.9992%) was sourced from AP BAIF Gases Industry Co., Ltd., China. All materials were utilized directly without any purification.

### Fabrication of the NF-based humidity sensor

**Fig. 1:**
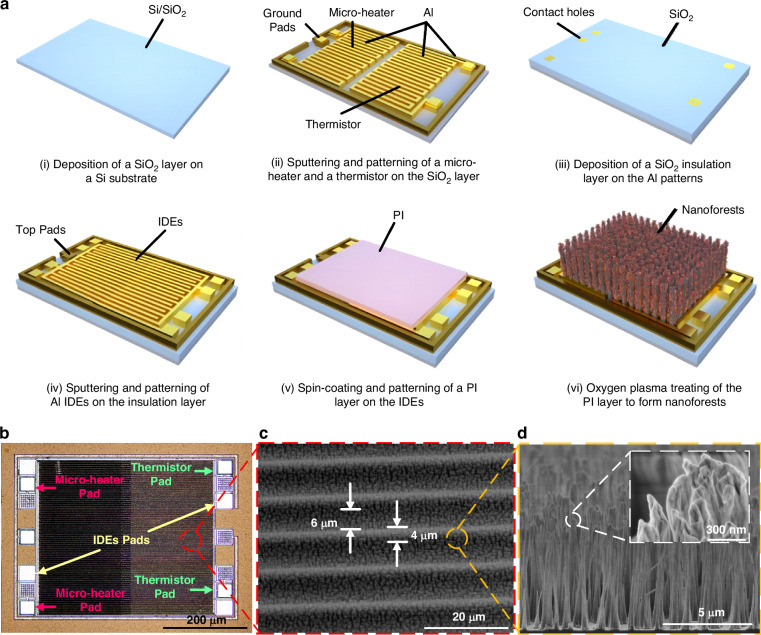
Illustrations for the humidity sensor. **a** Fabrication process for the nanoforest (NF)-based humidity sensor. **b** An optical image of the humidity sensor. **c**, **d** SEM images of NFs on the device

Figure 1a illustrates the fabrication process of the NF-based humidity sensor. The starting substrate is a Si wafer with a SiO_2_ insulation layer. On this substrate, a micro-heater, a thermistor and ground Pads are prepared simultaneously by patterning an Al layer on the SiO_2_ layer. Subsequently, another SiO_2_ insulating layer is deposited, on top of which a second Al layer is patterned to form the IDEs. A PI layer, approximately 8 μm thick, is then spin-coated onto the IDEs, followed by a time-controlled plasma treatment under an O_2_ plasma atmosphere, characterized by an O_2_ flow rate of 50 sccm, a radio frequency power of 200 W, and for a period of 30 min.

### Characterization

To observe the humidity sensor, A high-resolution microscope (BX51M, Olympus, Japan) was employed to capture optical images. Additionally, a Hitachi electron microscope (SEM, S-5500, Japan) was used to characterize the images of the NFs. The real-time temperatures of the sensor during operation were measured using a handheld infrared temperature camera (UD30528B-B, Hikmicro, China). Ambient humidity and temperature were monitored using a calibrated commercial hygrometer and thermometer (TH:101B, Anymartha, China).

Performance measurements of the sensor were conducted at 25 °C under standard atmospheric pressure. A temperature-humidity control chamber (KP880, Kaiping, China) was utilized to maintain temperatures of 15 °C, 25 °C, and 35 °C, along with varying humidity conditions around the sensor. The output capacitance and resistance of the sensor were recorded using a 6.5-digit multimeter (DMM6500, Keithley, USA). For the selectivity tests, the humidity sensor was placed in a sealed bottle, where different gases of varying concentrations were introduced, and the sensor’s responses were collected. Heart rate during exercise was monitored using a smartwatch (WATCH FIT 2, Huawei, China).

### Respiratory monitoring module

The respiration monitoring module comprises three main components, a signal acquisition and processing unit utilizing a microcontroller unit (MCU, HC32L110C4UA from Huada Semiconductor Co., Ltd., China), a timer unit based on the LMC555CMMX from Texas Instruments Semiconductor Manufacturing Co., Ltd., USA, and a wireless signal transmission unit featuring a Bluetooth chip (CH9141BLE2U, from Nanjing Qinheng Microelectronics Co., Ltd., China). In this configuration, the MCU collects output signals from the humidity sensor, converts the analog signals to digital form via its built-in ADC converter, and compensate for any temperature drift in the device. The Bluetooth wireless transmission unit sends the digital signals to a data processing terminal, which can be either a computer or a smartphone, while the timer unit assists in calculating the sensor capacitance. The respiration monitoring module operates a constant voltage supply of 3 V.

## Results and discussion

### Characterization of the humidity sensor

Figure 1b displays an optical image of the prepared humidity sensor. In this sensor, the micro-heater and its corresponding Pads are located on the left side, while the thermistor and its Pads are situated on the right side. The slight color difference between these two areas can be attributed to positional deviations between the overlapped IDEs and either the micro-heater or the thermistor. Additionally, the NFs in this sensor are distributed within an area of 850 μm × 820 μm, which covers the entire region of the IDEs. Figure 1c, d present SEM images of the NFs on the device. As shown in Fig. 1c, the NFs are distributed both on the IDEs and within the gaps between the IDE combs, where the width of each IDE comb is 4 μm, and the gap between them is 6 μm. Figure 1d provides a magnified view of the tops of the NFs, revealing numerous gaps and tiny pores, each measuring in the tens of nanometers. These features create ample active sites and space for the adsorption of water molecules.

### Performance of the NF-based humidity sensor

**Fig. 2:**
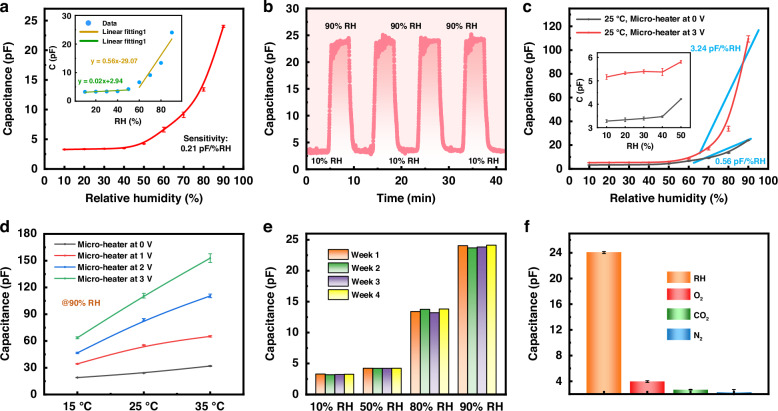
Performance of the NF-based humidity sensor. **a** Capacitance readout over a large RH range at 25 °C. **b** Continuous response and recovery curve of the sensor at 25 °C. **c** Capacitance responses of the sensor with and without a voltage applied to the micro-heater. **d** Capacitance curves of the device applied with different heating voltages. **e** Stability of the sensor at different RH conditions at 25 °C. **f** Gas selectivity of the sensor at 25 °C. The error bars of the data in **a**, **c**, **d**, **f** denote the standard deviation of three measurements

After the preparing of this novel sensor, its humidity sensing performance was systematically investigated. Prior to measurements, the environmental humidity at 25 °C was assessed using both a commercial hygrometer and the prepared sensor. As indicated in Fig. S1, the response of the humidity sensor in air is 3.4 pF, corresponding to approximately 40% RH. Subsequently, the capacitance responses of the sensor to varying RH conditions in the range of 10% to 90% RH were recorded, as illustrated in Fig. 2a. The data show that the capacitance of the device increases significantly with rising RH; at the 40% RH condition, the capacitance measures 3.47 pF, which is consistent with the environment humidity testing result. Notably, the sensor demonstrates high sensitivity of up to 0.56 pF/%RH when the RH exceeds 60%, indicating that the device is particularly suitable for detecting high humidity levels. Given that repeatability is a critical parameter for humidity sensors, we investigated the performance repeatability of the sensor from 10% to 90% RH at 25 °C, with the results presented in Fig. 2b. Overall, the output waveforms of the four cycles show little variation, indicating that the sensor possesses good dynamic stability.

To provide a comprehensive perspective, applying a voltage to the micro-heater also raises the temperature. This increase in voltage can enhance the output capacitance of the humidity sensor. At room temperature, a voltage of 3 V applied to the micro-heater elevates the sensor’s surface temperature to 57.1 °C (as shown in Fig. S2, captured using an infrared camera). The dielectric constant of humidity-sensitive materials exhibits a positive correlation with temperature. Furthermore, the dielectric constant is positively correlated with the capacitance value, leading to an increase in the device’s response as temperatures rise. A detailed discussion of the relevant theory can be found in the supplementary material. Consequently, the device’s sensitivity under high RH conditions increases by 5.8 times, changing from 0.56 pF/%RH to 3.24 pF/%RH, as illustrated in Fig. 2c. The recovery time for the sensor was assessed by reducing the RH from 80% to 30%, demonstrating a recovery time of 2.2 s (see Fig. S3).

To further investigate the impact of temperature on the sensor’s responses, we tested the device’s output capacitances at 90% RH while varying the applied voltage to the micro-heater. During this experiment, the ambient temperature ranged from 15 to 35 °C, and output capacitances are illustrated in Fig. 2d. Notably, the applied voltage shows a linear relationship between the ambient temperature (15–35 °C) and the sensor’s surface temperature (as shown in Fig. S4). With an applied voltage of 3 V, the surface temperature of the device increases by approximately 32 °C, resulting in significantly higher capacitances at each ambient temperature compared to those measured without an applied voltage (represented by the black line in Fig. 2d). Additionally, a larger applied voltage results in higher output capacitance. Furthermore, higher applied voltages correspond to greater output capacitances, underscoring that elevated sensor temperatures enhance device sensitivity.

Given the significant influence of surface temperature on the sensor’s response, it is crucial to monitor the surface temperature during operation. While an infrared (IR) camera can measure temperature, its use can be impractical. Therefore, we employed a built-in thermistor for temperature measurements. Figure S5 illustrates the linear relationship between the thermistor’s resistance and temperature. By utilizing this thermistor alongside the capacitance output of the sensor at various temperatures, we can effectively compensate for temperature drift in the device.

For humidity sensors intended for wearable devices, long-term stability is a critical parameter. To assess this stability, we recorded the capacitance output of the sensor after several weeks of storage. As depicted in Fig. 2e, the sensor maintains its original output at 25 °C over time, indicating excellent long-term stability. Additionally, during respiration, other gases accompany exhaled air; thus, it is essential to evaluate the device’s response to these gases, referred to as specificity detection. Figure 2f shows that the sensor exhibits high selectivity for water vapors in comparison to other interference gases, including O_2_, CO_2_ and N_2_, making it well-suited for respiratory monitoring applications.

### Sensing mechanism of the NF-based humidity sensor

**Fig. 3:**
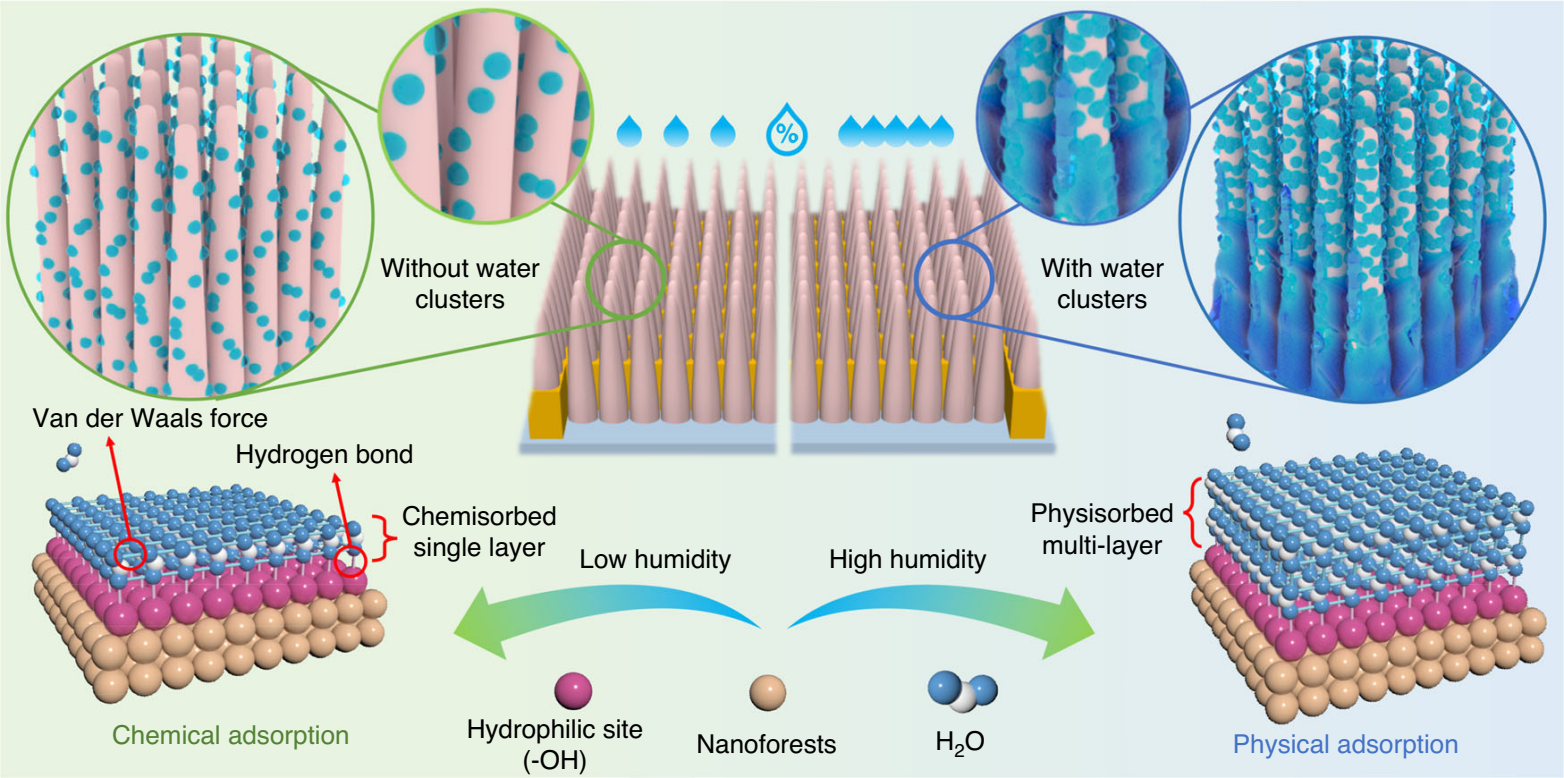
Humidity-sensing mechanism of the nanoforests. Schematic of water molecule adsorption mechanism surrounding the NFs at different humidity conditions

The performance of the NF-based humidity sensor indicates commendable humidity-sensing capabilities. This efficiency largely stems from the presence of numerous hydrophilic groups, such as carboxyl (–COOH) and hydroxyl (–OH), on their surfaces, which can form hydrogen bonds with surrounding water molecules^36^^.^ The large surface area of the NFs offers many active sites for water molecule adsorption. As shown in Fig. 3, during the adsorption process, water molecules first adhere to the hydrophilic NF surfaces through hydrogen bonding, creating a chemisorption layer. As RH increases, additional water molecules integrate with this chemisorption layer, resulting in multilayers of water molecules that are held together by Van der Waals forces, thereby forming a physisorption layer^37^. Importantly, while the chemisorption layer can accommodate a limited number of water molecules due to restricted active sites on the NFs, the physisorption layer permits multiple layers of water molecules, leading to a significant increase in the sensor’s capacitance output. This phenomenon explains the observed turning point in each capacitance response curve, resulting in different sensitivities for the sensor in low and high RH region. Additionally, capillary condensation occurs within the pores of the NFs, and because the pores vary in size on a nanoscale, capillary condensation can take place as RH conditions fluctuate widely. The presence of a humidity gradient drives condensed liquid to flow toward areas of lower humidity caused by the hydrophilic nature of the NFs’ surfaces, facilitating liquid movement along the surfaces. For instance, during a rising RH state, fewer water molecules may be present at the bottom, leading to the likelihood of condensed liquid accumulating there. Conversely, during a falling RH state, the top surface of the NFs become relatively deficient in water, prompting liquid movement upward. Moreover, within any given humidity environment, higher temperatures yield larger saturated absolute humidity levels, indicating that more water molecules are adsorbed onto the moisture-sensitive material within a unit volume of air. Higher temperatures also enhance the chemical activity of water molecules, promoting their binding with active groups on the NFs. Additionally, rising temperatures increase the diffusion velocity of water molecules, aiding their penetration into the narrow pores and gaps of the NFs^38,39^. This rapid diffusion enhances the probability of intermolecular collisions, leading to the agglomeration of water molecules into clusters, further boosting the sensitivity of the devices. Consequently, the sensor demonstrates higher sensitivity and shorter recovery times compared to previously reported humidity sensors, as detailed in Table S1 of the Supplementary Information^29,31,40–45^.

### Respiratory signal monitoring

**Fig. 4:**
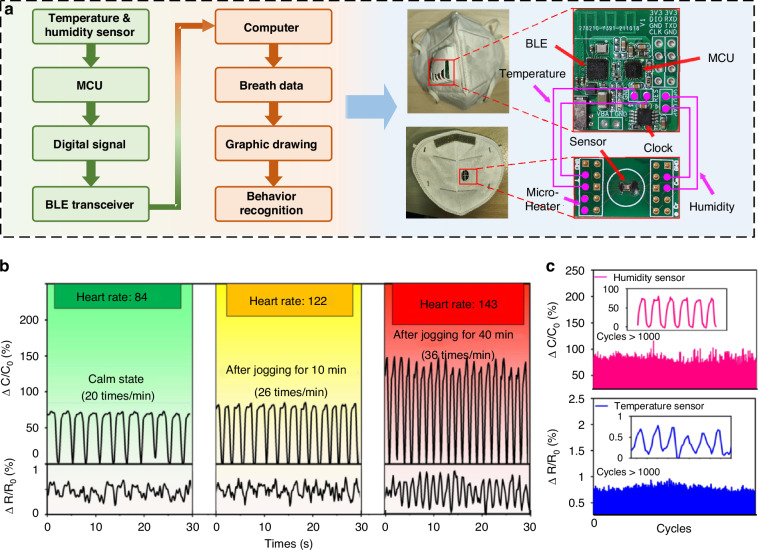
Scheme of a wireless system for respiratory data acquisition and transmission, where the sensor operates at 57.1 °C. **a** Flow chart for the behavior recognition process, and photographs of a mask inserted with a humidity sensor and a data processing module. **b** Humidity and temperature signals corresponding to different breathing states. **c** Output curves of the sensor over 1000 consecutive respiration cycles

With high sensitivity, long-term stability and good selectivity, the sensor is further utilized for behavior recognition by monitoring respiration in real time. The flow chart of the behavior recognition system is illustrated in Fig. 4a. In this study, the sensor is integrated into the ventilation valve of a mask to collect respiratory signals, including temperature and RH data during breathing. These signals are transmitted to a computer via a Bluetooth transmission module. To enhance the distinct characteristics of the collected signals, the micro-heater was supplied with a voltage of 3 V during the experiment.

To accurately demonstrate the fluctuation of humidity in respiration, the relative variation rate of capacitance is defined as

1 $${\rm{Response}}\left(C\right)=\,\frac{{C}_{{\rm{R}}}-{C}_{0}}{{C}_{0}}=\,\Delta C/{C}_{0}$$

Where ${C}_{{\rm{R}}}$ is the output capacitance of the humidity sensor and ${C}_{0}$ is the initial capacitance at ambient humidity. Similarly, the relative variation rate of resistance is defined as

2 $${\rm{Response}}\left(R\right)=\,\frac{{R}_{{\rm{R}}}-{R}_{0}}{{R}_{0}}=\,\Delta R/{R}_{0}$$

Here, ${R}_{{\rm{R}}}$ is the recorded resistance of the thermistor and ${R}_{0}$ is the initial resistance at ambient temperature.

To investigate the system’s ability to discriminate daily behaviors, respiratory data, including Response (*C*) and the Response (*R*) of a volunteer in three different states related to jogging, were collected, as illustrated in Fig. 4b. These states include the resting state (~ 20 breaths/min), the state after 10 min of jogging (~ 26 breaths/min), and the state after 40 min of jogging (~ 36 breaths/min). The corresponding heart rates collected by a smartwatch are indicated in Fig. 4b. The results demonstrate that the sensor can distinguish different behavioral states through the frequencies and amplitudes of the humidity and temperature signals, laying the foundation for subsequent recognition. Before practical use, the respiratory monitoring module’s ability to operate continuously and steadily was assessed, with stable curves of the sensor recorded over 1000 consecutive respiration cycles shown in Fig. 4c.

### Behavior recognition based on the humidity sensor

**Fig. 5:**
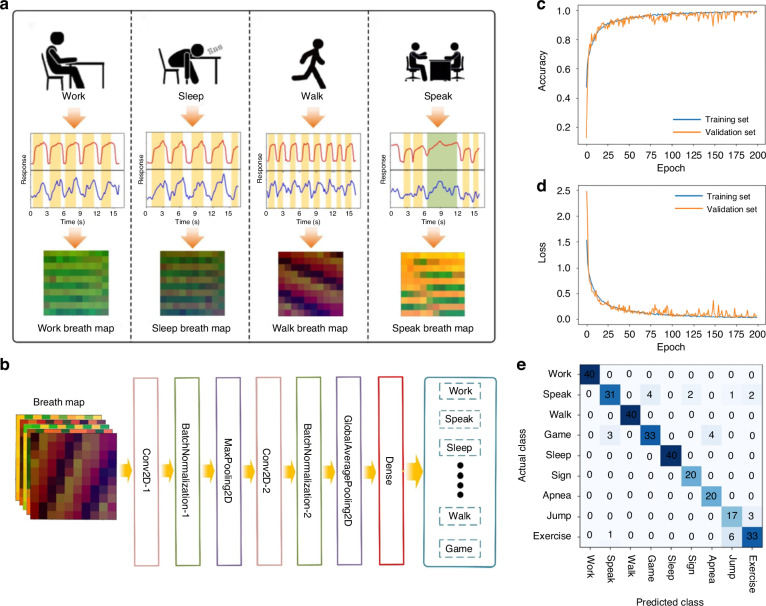
The process of training and validating a high accuracy model using respiratory data, where the sensor operates at 57.1 °C. **a** Data processing of four typic behavioral states. **b** Framework of the training process. **c**, **d** Classification accuracy and loss curves of the model. **e** Confusion matrix of the actual class and predicted class for recognizing nine behavior states

Building on the features of the respiratory monitoring module, a convolutional neural network (CNN) is developed to distinguish respiratory information and corresponding behaviors. To achieve this recognition, the time-frequency characteristics of respiratory humidity and temperature are analyzed. Humidity, temperature, and time information correspond to RGB pixels to construct a two-dimensional map. In this work, 100 data were converted into a two-dimensional map (with 10 × 10 pixels), which served as the input parameters for the neural network model (shown in Fig. S6). Figure 5a demonstrates the original response data obtained by the sensor alongside the RGB data of four typic behavioral states. For analysis, we selected nine common behaviors (Work, Speak, Walk, Play electronic games, Sleep, Sigh, Apnea, Jump and Exercise). According to the respiratory waveforms (shown in Fig. S7), the respiratory patterns for Work, Walk, Sleep and Exercise are relatively orderly. Notably, the respiratory frequency and Response (*R*) for Exercise are significantly higher, measuring 36 times/min and exceeding 100%, respectively. Among the other three states, the highest Response (*R*) is observed in Walk, while Work shows the lowest value. This discrepancy is assumed to stem from variations in body temperature across different behavioral states. Furthermore, the other five respiratory states exhibit clear characteristics; for instance, the humidity signal remains constant during apnea but dramatically increases during sighing. In summary, the respiratory data for the nine behaviors contain extractable characteristics.

The recognition of behaviors is implemented using Python 3.8 tools. The Numpy library processes the breath data, while the Scikit-learn library is employed to train and validate the behavior recognition model. Figure 5b illustrates the framework of the training and validating process, which consists of a convolutional layer, a batch normalization layer, a pooling layer, and a dense layer. To develop a high accuracy intelligent recognition model, a dataset containing 200 maps for Sigh, Apnea, Jump and 400 maps for the other six states were prepared. The data were divided into training, validation, and testing classes with a ratio of 7:2:1. These data classes were then trained to build the model, with features from the nine breath maps extracted and optimized through ConV2D for preliminary feature extraction, Relu to enhance feature expression, BatchNomalization to improve training efficiency, and MaxPooling2D to reduce the output dimension of the convolution layer and decrease computational load. Afterward, the features of the nine states were categorized by the classifier dense layer. Following training, a model with high recognition accuracy and a low loss rate for nine behaviors was established. Figure 5c, d illustrate the classification accuracy and loss curves of the model as a function of the number of training sessions. Notably, once the number of training sessions reached 75, the classification accuracy for both the training and validation classes rapidly increased to 96.2%. These results indicate that, with continued learning iterations, the classification accuracy and loss curves for the validation class closely mirrored those of the training class, suggesting that there is no overfitting issue in the optimized model.

Based on the developed algorithm, the classification accuracy for five breathing states-Work, Walk, Sleep, Sigh, and Apnea-reaches 100%, as shown in Fig. 5e. This high accuracy is attributed to the distinct characteristics of these breathing states. However, the Jump state, being a transient form of Exercise, shares some breathing characteristics with Exercise, which can occasionally lead to misidentifications by the machine learning algorithm. Similarly, the behavior of Speak may occur during the Game state, resulting in occasional misjudgment of Speak as Game. By training with a larger dataset of respiratory data, the likelihood of such misjudgment can be reduced, thereby enhancing the accuracy of the behavior recognition model.

**Fig. 6:**
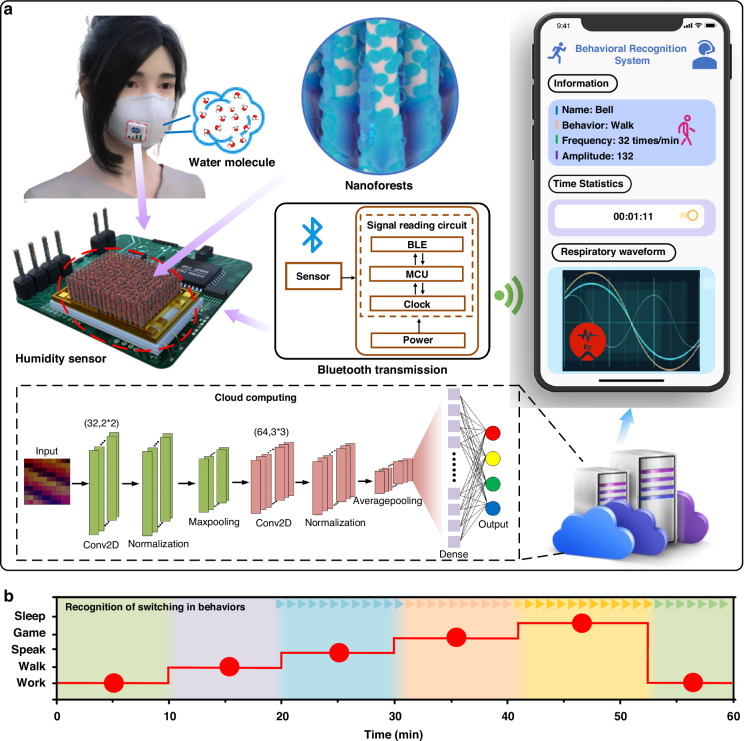
The behavior recognition system based on the novel humidity sensor and its potential applications in smart healthcare. **a** Schematic diagram of the behavior recognition system and its main components. **b** Continuous recognition of different behaviors in a period of time

To demonstrate the potential applications of the proposed behavior recognition system in advanced healthcare, Fig. 6a illustrates how respiratory states can be converted into real-time behavioral information, accessible anytime and anywhere using a smartphone. The wireless data transmission unit sends breathing data to the smartphone via Bluetooth, which then uploads the respiratory data to a cloud server through a network connection. Subsequently, the real-time breathing data are fed into the classification module through the server and compared with the trained high-accuracy model. Finally, these visualization results are displayed on a designed page realized through the applet function of WeChat on the smartphone. This integration of a powerful open platform and functional components, such as canvas drawing, cloud services, and “wxml” page design, enables real-time processing and display of information on mobile devices.

To validate our smart system, it is further applied to record volunteers’ behaviors in daily life. Figure S8 presents the information displayed on the smartphone, which accurately reflects the volunteers’ behavioral states (e.g. the Work state). The interface showcases data including respiratory frequency, amplitude, waveform, and time ratio, facilitating users in monitor their body conditions. With the continuous operation of the system, the monitoring and recognition of different behaviors can be conducted promptly and effectively, as illustrated in Fig. 6b. Additionally, the system can provide the proportion of different behaviors over this period, as displayed in Fig. S9. These results support the feasibility of such an intelligent system for daily healthcare.

## Conclusion

In conclusion, we have fabricated a novel humidity sensor based on NFs using a low-cost, CMOS-compatible process. By utilizing the extensive active sites for water molecule adsorption in the NFs, the fabricated sensor demonstrates remarkably high sensitivity, stability and selectivity. These features enable the sensor to be employed for behavior recognition through respiration detection. Furthermore, the development of a CNN-based algorithm has achieved a recognition accuracy of 96.2%. This real-time human behavior recognition system provides a promising application direction for the future development of the health electronics industry, encompassing the analysis of physical states in daily life and the medical care of hospital patients.

## Supplementary information

Supplementary Information(Clean Version) of MICRONANO-03803R
