# Supplementary material for: An intelligent humidity sensing system for human behavior recognition
Source: Microsyst Nanoeng. 2025 Jan 22;11:17. doi: 10.1038/s41378-024-00863-6 (PMC11751383; doi:10.1038/s41378-024-00863-6)
Supplement: Supplementary file 1 — Supplementary Information(Clean Version) of MICRONANO-03803R [file 41378_2024_863_MOESM1_ESM.docx]

**An** **Intelligent Humidity Sensing System for Human Behavior Recognition**

Huabin Yang ^1,2^, Qiming Guo ^1,2^, Guidong Chen ^1,3^, Yuefang Zhao ^1,2^, Meng Shi ^1,2^, Na Zhou ^1,2,*^, Chengjun Huang ^1,2^, and Haiyang Mao ^1,2,*^

^1^ Institute of Microelectronics of the Chinese Academy of Sciences, Beijing 100029, China

^2^ University of Chinese Academy of Sciences, Beijing 100049, China

^3^ BYD Auto Industry Company Limited, Shenzhen 518118, China

^*^ Corresponding authors:

E-mails: [maohaiyang@ime.ac.cn](mailto:maohaiyang@ime.ac.cn) (H. Mao), [zhouna@ime.ac.cn](mailto:zhouna@ime.ac.cn) (N. Zhou).

**
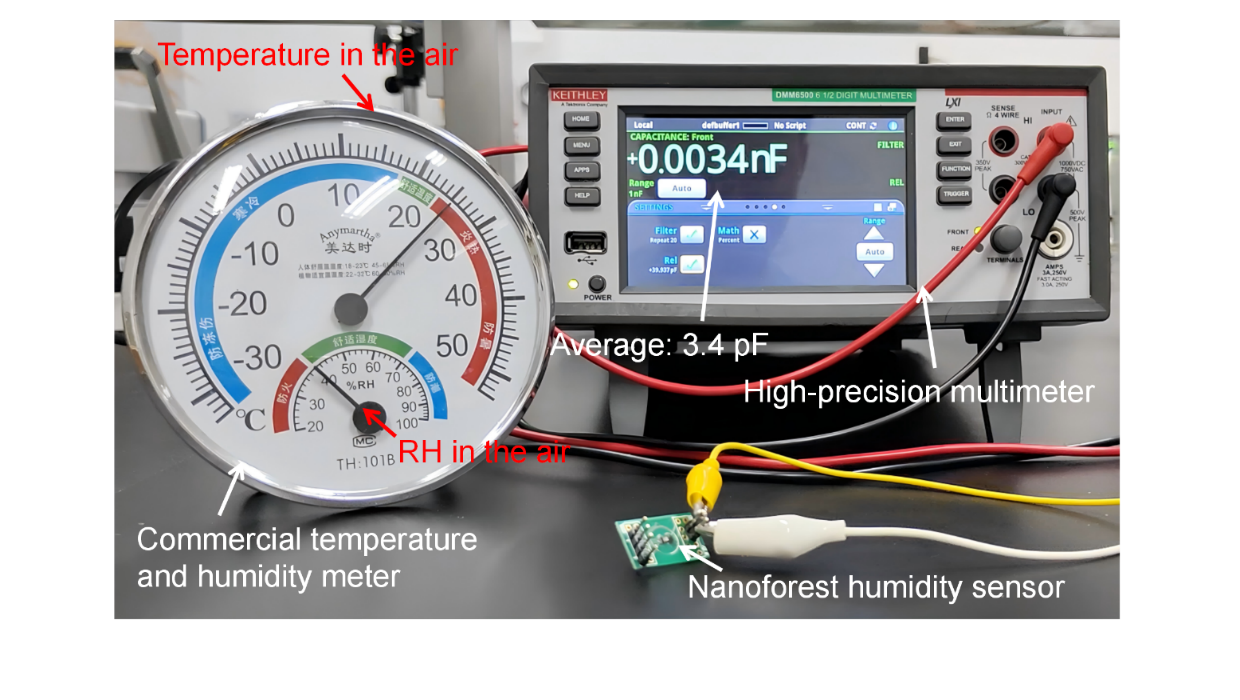
**

**Fig. S1.** Measuring the relative humidity response properties in air.

**
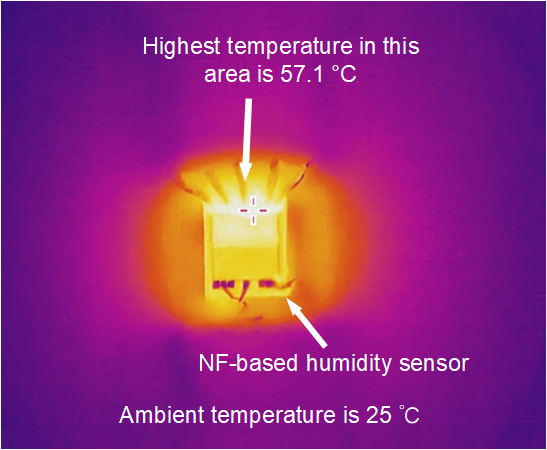
**

**Fig. S2.** Temperature distribution image of the sensor while its micro-heater is applied with a voltage of 3 V.


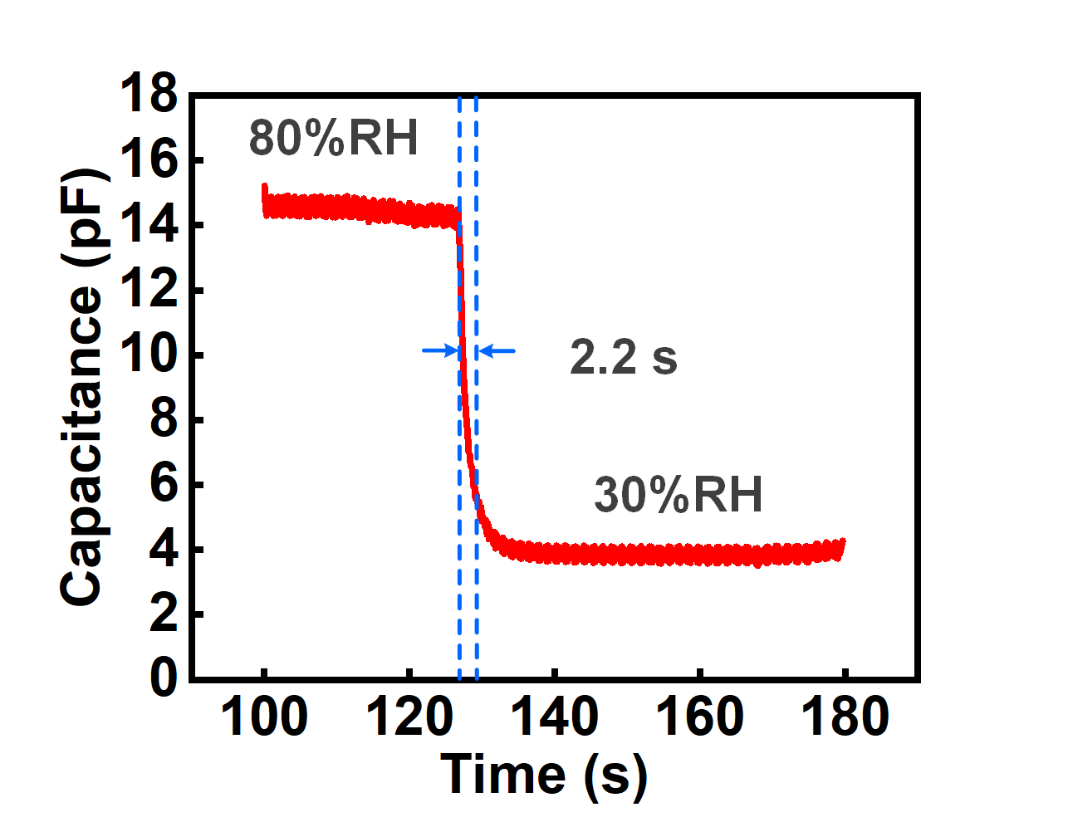


**Fig. S3** Recovery time of the nanoforest humidity sensor.


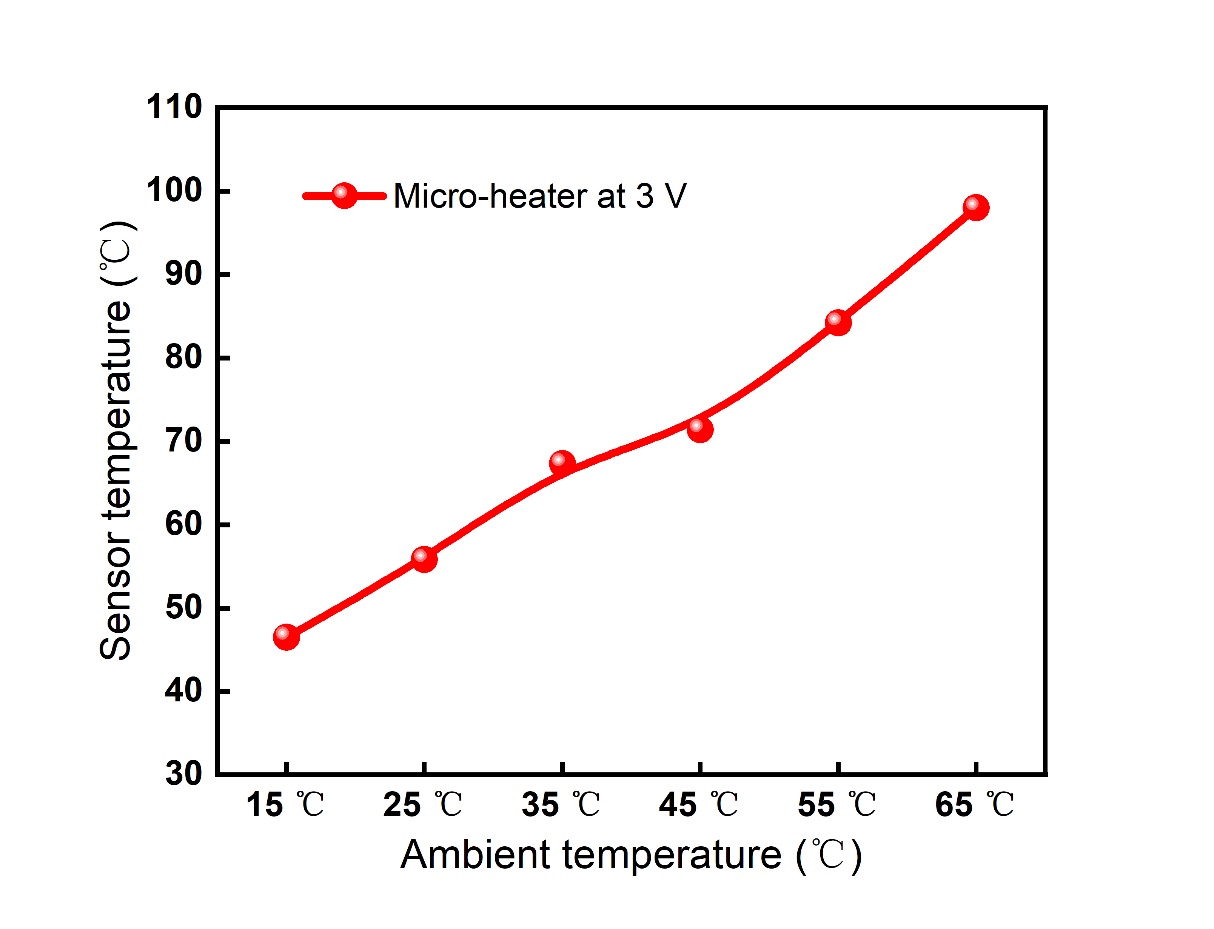


**Fig. S4.** The sensor surface-ambient temperature curve while its micro-heater is applied with a voltage of 3 V.

**
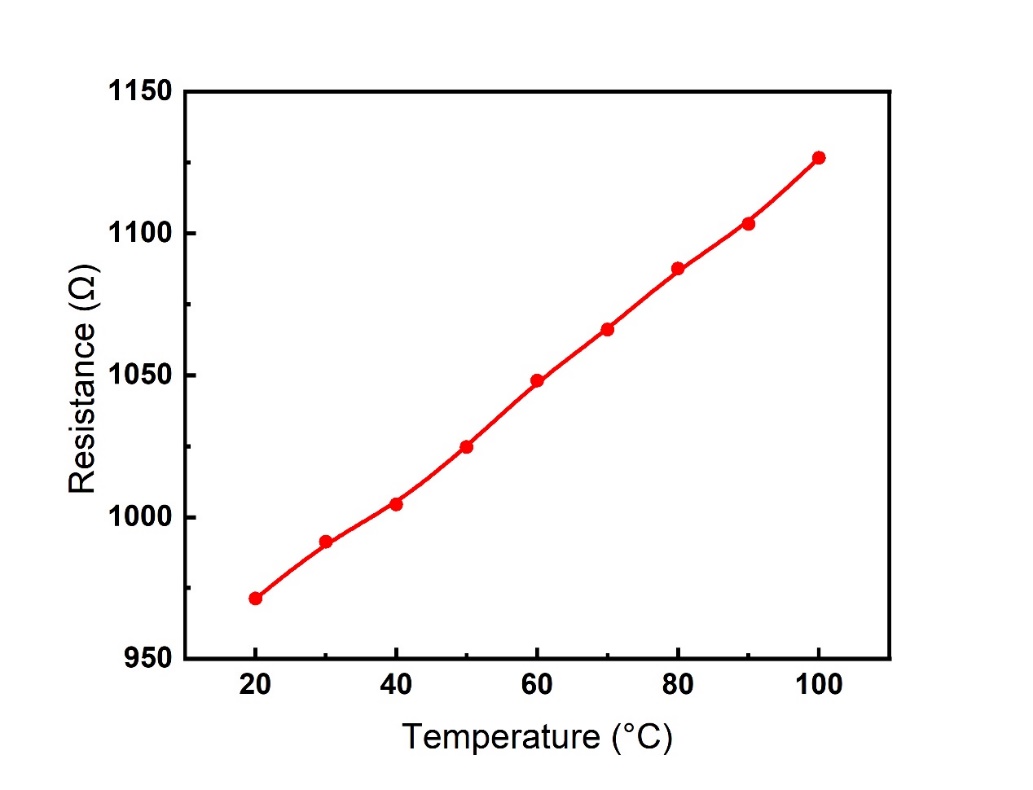
**

**Fig. S5.** The resistance-temperature response curve of the thermistor.

**Principle of Humidity Sensor Sensitivity to Heat Increase:**

The Looyenga semi-empirical formula is commonly employed to establish the relationship between the dielectric constant and relative humidity, expressed as:

$$\varepsilon=[\gamma\left( \varepsilon_{w}^{\frac{1}{3}}-\varepsilon_{p}^{\frac{1}{3}} \right)+\varepsilon_{p}^{\frac{1}{3}}]^{3}$$

Here, $\varepsilon$ represents the dielectric constant of sensitive material after moisture absorption, $\varepsilon_{w}$ is the dielectric constant of water molecules, $\varepsilon_{p}$ denotes the dielectric constant of sensitive material at a relative humidity of 0, and $\gamma$ indicates the volume ratio of the sensitive material film after moisture absorption.

The dielectric constant of water, $\varepsilon_{w}$, is temperature-dependent and is expressed by the following equation:

$$\varepsilon_{w}=78.54\{1-4.6\times{10}^{-4}\left( T-298 \right)+8.8\times{10}^{-6}\left( T-298 \right)^{2}\}$$

To calculate the volume ratio $\gamma$ of the sensitive material absorbing water vapor, the empirical equation for solid adsorption is employed:

$$\gamma=\gamma_{m}\Phi(T)x^{\Psi(T)}$$

The temperature dependence of the adsorption coefficient is given by:

$$\Phi\left( T \right)=1-\alpha_{0}(T-T_{0})$$

The temperature dependence of the water dielectric constant follows the equation:

$$\Psi\left( T \right)=\Psi_{0}\frac{1-\alpha_{1}\left( T-T_{0} \right)+\alpha_{2}\left( T-T_{0} \right)^{2}}{1+\beta_{1}exp[\beta_{2}(T-T_{0})]}$$

From the above equations, it is evident that the dielectric constant varies with temperature. As the temperature increases, the dielectric constant also rises, leading to enhanced output sensitivity.

**
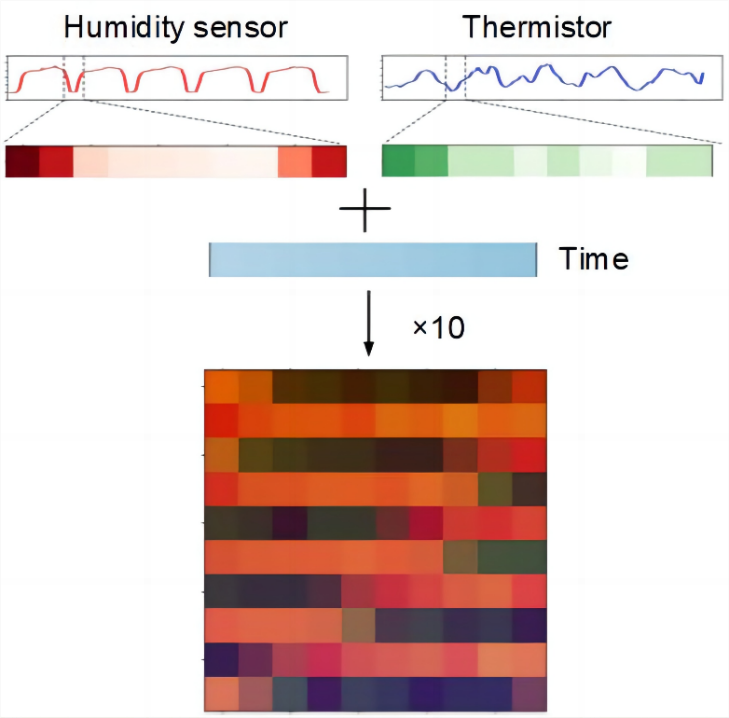
**

**Fig. S6.** Respiratory data fusion algorithm.


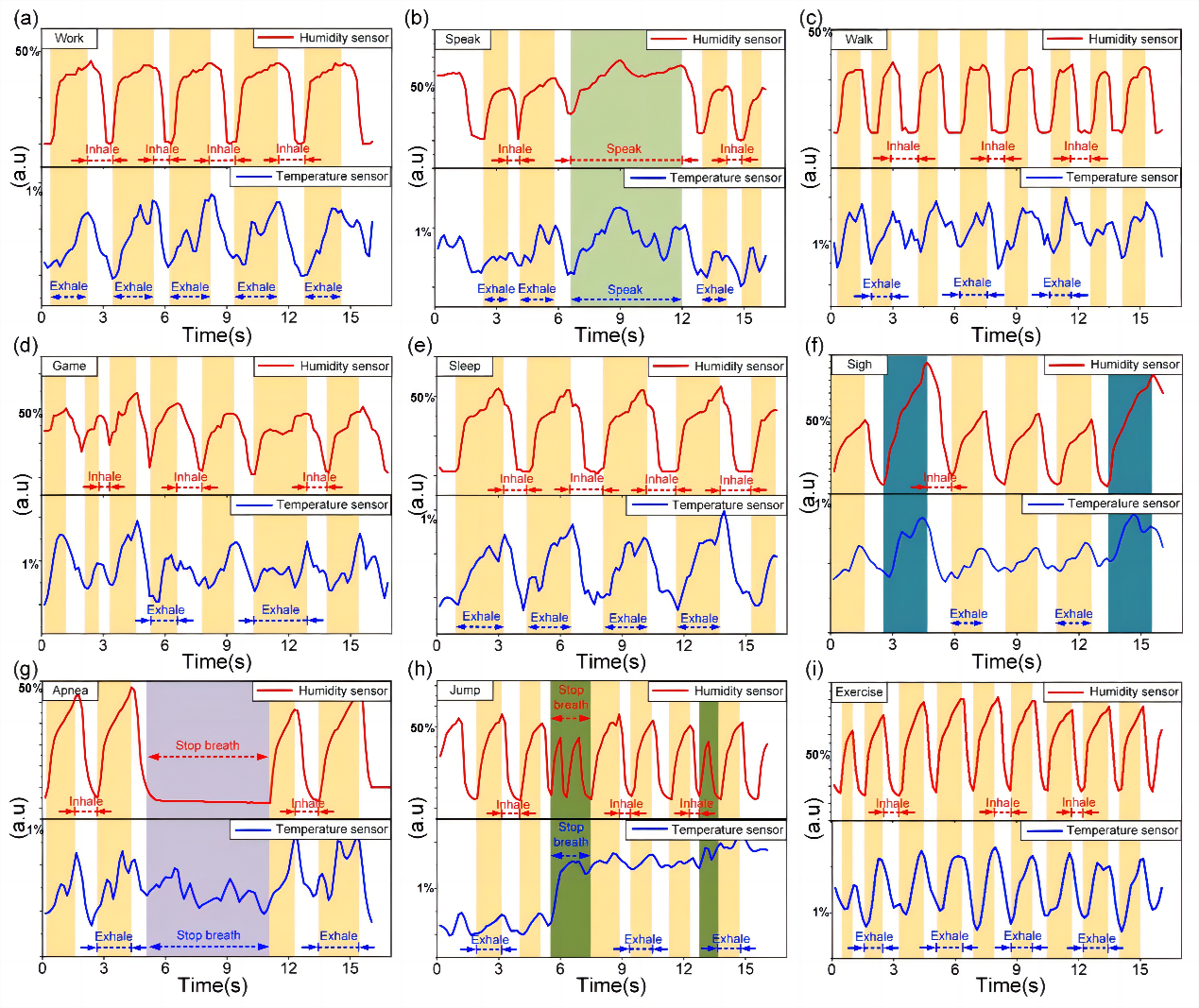


**Fig. S7.** The capacitance and resistance curves of the sensor during continuous respiratory monitoring under nine different human behaviors.


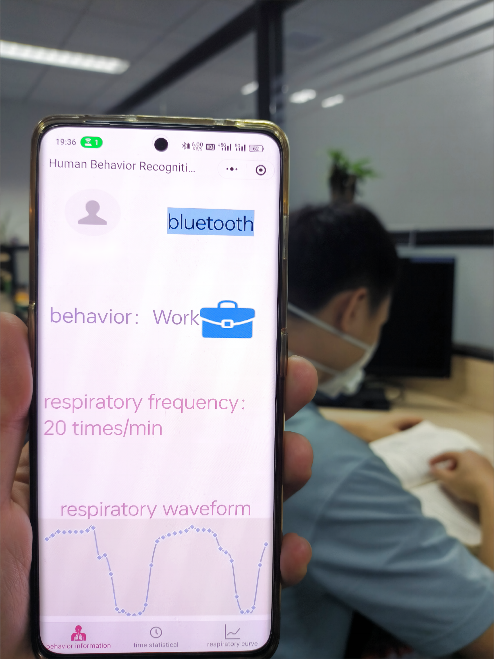


**Fig. S8.** Human behavior recognition for the state of Work.

**
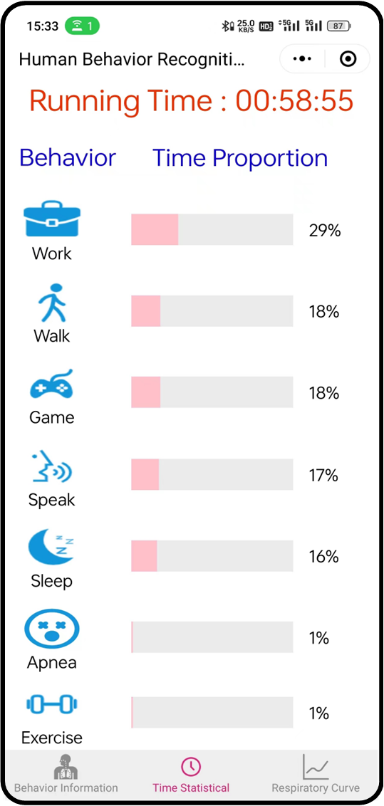
**

**Fig. S9.** Time statistics for five different behaviors in one hour.

Table S1. Performance comparison of humidity sensors using different sensing materials

| **Materials** | **Sensitivity** | **Recovery time** | **Footprint** | **Ref** |
| --- | --- | --- | --- | --- |
| MoS_2_ | 0.002 mW/%RH | 2.395 s | 0.27 mm^2^ | [1] |
| GO | 25.3 kHz/%RH | 10 s | π 4.5^2^ mm^2^ | [2] |
| PI nanostructure | 0.077pF/%RH | 7 s | 2.3 mm^2^ | [3] |
| P(VDF-TrFE) nanocone | 0.0015pF/%RH | 3.43 s | 75 mm^2^ | [4] |
| AAO | 41.6nF/%RH | 11 s | 525 mm^2^ | [5] |
| Polymer/TiO_2_ | 1.24pF/%RH | 25 s | 20 mm^2^ | [6] |
| ZnO NRs/WS2 | 0.1pF/%RH | 25.67 s | - | [7] |
| paper | 2 pF/%RH | 41 s | 5 mm^2^ | [8] |
| Nanoforest | 3.24pF/%RH | 2.2 s | 0.98 mm^2^ | This work |

1. Du, B. et al. MoS_2_-based all-fiber humidity sensor for monitoring human breath with fast response and recovery. *Sens. Actuators, B* **251**, 180-184 (2017).
2. Le, X. et al. Surface acoustic wave humidity sensors based on uniform and thickness controllable graphene oxide thin films formed by surface tension. *Microsyst. Nanoeng.* **5**, 36 (2019).
3. Lee, H., Lee, S., Jung, S., Lee, J. Nano-grass polyimide-based humidity sensors. *Sens. Actuators, B* **154**, 2-8 (2011).
4. Niu, H. et al. Ultrafast-response/recovery capacitive humidity sensor based on arc-shaped hollow structure with nanocone arrays for human physiological signals monitoring. *Sens. Actuators, B* **334**, 129637 (2021).
5. Chung, C. K., Ku, C. A., Wu Z. E. A high-and-rapid-response capacitive humidity sensor of nanoporous anodic alumina by one-step anodizing commercial 1050 aluminum alloy and its enhancement mechanism. *Sens. Actuators, B* **343**, 130156 (2021).
6. Tian, Q. et al. High-performance porous MIM-type capacitive humidity sensor realized via inductive coupled plasma and reactive-ion etching. *Sens. Actuators, B* **258**, 704-714 (2018).
7. Muhammad, A. D., Farah, F., Cuk I., Vivi F. The enhanced performance of capacitive-type humidity sensors based on ZnO nanorods/WS_2_ nanosheets heterostructure. *Sens. Actuators, B* **310**, 127810 (2020).
8. Yang, Q. et al. Capillary condensation under atomic-scale confinement. *Nature* **588**, 250-253 (2020).
